# Second Sight

**Published:** 1891-03

**Authors:** 


### CHILDREN.

### SECOND SIGHT.

Some forty years ago, in North-eastern Ohio, before the time of railroads and hotels, says a writer in the *Globe-Democrat*, a woman and her husband came through that section, travelling to the West. They stopped at my father's farm and stayed over night, and in the course of the evening's conversation it came out that the wife, a fresh faced country woman, possessed the gift called by the Scotch second sight, and which is now generally called clairvoyance.

She did not go into a trance to see things. "I just put my hand before my eyes and look through my head," said she. "I cannot explain it any other way. My eyes are shut, but they seem to see right through my forehead. I see them not as I see with my eyes open, but as if I looked through a narrow opening and could only see one object at a time, but that very plainly."

Her gift was destined to be of profit to my father's family, for before the evening was over my mother begged her to give us a test.

"Look and try to see something that concerns us," she said.

The woman covered her eyes, bent her head forward, and finally said: "I see an old gentleman, very old and very feeble, dressed in blue broadcloth with brass buttons and wearing a ruffled shirt. On his arm is an old lady in a yellow chintz gown, with brown figures in it. Something ails her throat; it is muffled up."

And so she continued, giving an accurate description of my grandparents, one of whom had died three years before and the other within a few months, and describing the dress they most commonly wore down to the minutest details, which could not have been given her by the neighbors, for they had even been forgotten by the family, and were only remembered when she mentioned them.

"Can you find things that are lost?" asked my mother. "May be you can tell us who stole Simon's $100."

The woman drew a long, quiet breath, and still with covered eyes, said: "It was not stolen, I think."

"I'm sure it was," said father; "I know right where I put it, and when I looked for it it was gone."

The woman continued, however, saying: "You leave this house by a south door. You go down a path more than fifty steps, then you come to the barn. No, you do not stop there. Oh, here it is, you stop. I think it is a chicken house. Here is a nest, and in the straw lies the money."

Father had been lighting his lantern as she spoke, and had it ready as she finished, and hurried down to the hen-house—we children racing after him. Sure enough, there in a deserted nest, among broken and addled eggs, lay the roll of bills.

"I had it in my pocket when I was trying to set that pesky yellow hen," said father, "and she struggled and fought so that I must have dropped it out in the melee."

When we got back to the house the woman was chatting pleasantly with mother. She would take nothing for her services, saying that the gift was from God, and if she were paid for making use of it, it would leave her. However, we prevailed on her to spend a week with us, and many old persons now living who then lived in that section, still remember her and some of the wonderful tests she gave in finding lost articles and telling the whereabouts of those absent.

